# Supplementary material for: Individual retrotransposon integrants are differentially controlled by KZFP/KAP1-dependent histone methylation, DNA methylation and TET-mediated hydroxymethylation in naïve embryonic stem cells
Source: Epigenetics Chromatin. 2018 Feb 26;11:7. doi: 10.1186/s13072-018-0177-1 (PMC6389204; doi:10.1186/s13072-018-0177-1)
Supplement: Supplementary file 11 — Additional file 11. Pattern analysis. [file 13072_2018_177_MOESM11_ESM.zip › Patterns analysis/patterns.html]

Pattern analysis


# All the GAGA patterns

Pattern no | Png | N pattern size | Fam enrichement html | Fam enrichement xls | Pattern content xls (ERE) | Pattern content bed (ERE) |
| p01\_1 |  | N = 88 | html | xls | xls | bed |
| p01\_2 |  | N = 20 | html | xls | xls | bed |
| p02\_1 |  | N = 265 | html | xls | xls | bed |
| p02\_2 |  | N = 388 | html | xls | xls | bed |
| p03\_1 |  | N = 15 | html | xls | xls | bed |
| p03\_2 |  | N = 15 | html | xls | xls | bed |
| p04 |  | N = 40 | html | xls | xls | bed |
| p05 |  | N = 41 | html | xls | xls | bed |
| p06 |  | N = 160 | html | xls | xls | bed |
| p07 |  | N = 202 | html | xls | xls | bed |
| p08 |  | N = 56 | html | xls | xls | bed |
| p09 |  | N = 3226 | html | xls | xls | bed |
| p10 |  | N = 94 | html | xls | xls | bed |
| p11 |  | N = 952 | html | xls | xls | bed |
| p12\_1 |  | N = 115 | html | xls | xls | bed |
| p12\_2 |  | N = 94 | html | xls | xls | bed |
| p13 |  | N = 10 | html | xls | xls | bed |
| p14 |  | N = 45 | html | xls | xls | bed |
| p15 |  | N = 5 | html | xls | xls | bed |
| p16 |  | N = 33 | html | xls | xls | bed |
| p17 |  | N = 135 | html | xls | xls | bed |
| p18 |  | N = 194 | html | xls | xls | bed |
| p19\_1 |  | N = 55 | html | xls | xls | bed |
| p19\_2 |  | N = 83 | html | xls | xls | bed |
| p20\_1 |  | N = 278 | html | xls | xls | bed |
| p20\_2 |  | N = 185 | html | xls | xls | bed |
| p21\_1 |  | N = 89 | html | xls | xls | bed |
| p21\_2 |  | N = 11 | html | xls | xls | bed |
| p22 |  | N = 20 | html | xls | xls | bed |
| p24 |  | N = 4698 | html | xls | xls | bed |
| p25\_1 |  | N = 507 | html | xls | xls | bed |
| p25\_2 |  | N = 100 | html | xls | xls | bed |
| p26\_1 |  | N = 39 | html | xls | xls | bed |
| p26\_2 |  | N = 37 | html | xls | xls | bed |
| p27\_1 |  | N = 16 | html | xls | xls | bed |
| p27\_2 |  | N = 40 | html | xls | xls | bed |
| p28\_1+p25\_2+p31\_4 |  | N = 235 | html | xls | xls | bed |
| p28\_1+p25\_2 |  | N = 180 | html | xls | xls | bed |
| p28\_1 |  | N = 80 | html | xls | xls | bed |
| p28\_2 |  | N = 17 | html | xls | xls | bed |
| p29\_1+p49+p31\_2 |  | N = 718 | html | xls | xls | bed |
| p29\_1 |  | N = 346 | html | xls | xls | bed |
| p29\_2 |  | N = 426 | html | xls | xls | bed |
| p30 |  | N = 131 | html | xls | xls | bed |
| p31\_1 |  | N = 58 | html | xls | xls | bed |
| p31\_2 |  | N = 213 | html | xls | xls | bed |
| p31\_3 |  | N = 139 | html | xls | xls | bed |
| p31\_4 |  | N = 55 | html | xls | xls | bed |
| p32 |  | N = 67 | html | xls | xls | bed |
| p33 |  | N = 86 | html | xls | xls | bed |
| p34 |  | N = 80 | html | xls | xls | bed |
| p35 |  | N = 75 | html | xls | xls | bed |
| p36 |  | N = 87 | html | xls | xls | bed |
| p37 |  | N = 78 | html | xls | xls | bed |
| p38 |  | N = 1 | html | xls | xls | bed |
| p39\_1 |  | N = 14 | html | xls | xls | bed |
| p39\_2 |  | N = 272 | html | xls | xls | bed |
| p40 |  | N = 27 | html | xls | xls | bed |
| p41\_1 |  | N = 92 | html | xls | xls | bed |
| p41\_2 |  | N = 34 | html | xls | xls | bed |
| p42 |  | N = 3 | html | xls | xls | bed |
| p43\_1 |  | N = 10 | html | xls | xls | bed |
| p43\_2 |  | N = 54 | html | xls | xls | bed |
| p44 |  | N = 3 | html | xls | xls | bed |
| p45 |  | N = 68 | html | xls | xls | bed |
| p46 |  | N = 50 | html | xls | xls | bed |
| p47 |  | N = 0 | html | xls | xls | bed |
| p48 |  | N = 4 | html | xls | xls | bed |
| p49 |  | N = 159 | html | xls | xls | bed |
| p50 |  | N = 9 | html | xls | xls | bed |
| p51\_1 |  | N = 10 | html | xls | xls | bed |
| p51\_2 |  | N = 9 | html | xls | xls | bed |
| p52 |  | N = 32 | html | xls | xls | bed |
| p53 |  | N = 11 | html | xls | xls | bed |
| p54 |  | N = 4 | html | xls | xls | bed |
 Pattern no | Png | N pattern size | Fam enrichement html | Fam enrichement xls | Pattern content xls (ERE) | Pattern content bed (ERE) |
